# LncRNA00518 promotes cell proliferation through regulating miR-101 in bladder cancer

**DOI:** 10.7150/jca.35710

**Published:** 2020-01-14

**Authors:** Kunpeng Wang, Long Ma, Jingyuan Tang, Qiu Yu, Yang Shen, Yunfei Wei, Chen Zhu, Zhonglei Deng, Wei Zhang

**Affiliations:** 1 Department of Urology, Lianyungang Clinical College of Nanjing Medical University, Lianyungang, 222061, China.; 2 Department of Urology, The First Affiliated Hospital of Nanjing Medical University, Nanjing, 210029, China.; 3 Department of Urology, Jiangsu Province Hospital of Chinese Medicine, Affiliated Hospital of Nanjing University of Chinese Medicine, Nanjing 210029, China

**Keywords:** Bladder cancer, Lnc00518, MiRNA-101, EZH2

## Abstract

The purpose of our study is to elucidate the expression of lncRNA00518 (lnc00518) in the bladder cancer, and its potential mechanism in regulating the development of bladder cancer. The expression of lnc00518 in bladder cancer tissues and cells was examined by qRT-PCR. Correlation between lnc00518 expression with clinicopathologic characteristics and prognosis of bladder cancer patients was analyzed. *In vitro* effects of lnc00518 on the cellular behaviors of bladder cancer cells were explored. Moreover, *in vivo* effect of lnc00518 was evaluated by subcutaneous tumorigenesis in nude mice. The possible miRNA targets of lnc00518 were predicted by bioinformatics and further confirmed by dual-luciferase reporter gene assay, RIP and rescue experiments. Lnc00518 was highly expressed in bladder cancer tissues and cells. Lnc00518 expression was correlated with TNM staging and histological grade of bladder cancer. Besides, the overall survival was lower in bladder cancer patients with high expression of lnc00518 relative to those with low expression. Overexpression of lnc00518 enhanced proliferative, invasive, migratory potentials and clonality, but shortened G0/G1 phase of bladder cancer cells. Lnc00518 knockdown obtained the opposite trends. *In vivo* experiments revealed that lnc00518 knockdown inhibited subcutaneous tumorigenesis in nude mice. QRT-PCR results indicated that lnc00518 expression was negatively correlated with miRNA-101 expression in bladder cancer cells. Through dual-luciferase reporter gene assay and RIP, we confirmed the binding between lnc00518 and miRNA-101. Furthermore, EZH2 was verified to be the target of miRNA-101. MiRNA-101 knockdown reversed the inhibitory roles of lnc00518 knockdown in proliferative, migratory and invasive potentials of bladder cancer cells. Lnc00518 is highly expressed in bladder cancer and can be served as a predictor of poor prognosis. Lnc00518 promotes the proliferative, invasive and migratory potentials of bladder cancer by upregulating EZH2 *via* competitively binding to miRNA-101.

## Introduction

Bladder cancer is a common malignancy, with globally 386,000 new cases per year 1. Based on histological classification, bladder cancer includes urothelial carcinoma, squamous cell carcinoma and adenocarcinoma. Among them, urothelial carcinoma is the major histological type, accounting for more than 95% of all subtypes of bladder cancer 2,3. Although the treatment of bladder cancer has greatly progressed, the 5-year survival, morbidity and mortality have not improved very well in the past decade, which may be explained by its complex pathogenesis 4,5. Molecular abnormalities have been considered as very important pathogenic factors of bladder cancer, and and relative researches in this area is still in the initial stage 6. Molecular diagnosis and targeted therapy are newly discovered approaches for bladder cancer treatment 7,8. Clarification of the pathogenesis of bladder cancer is important, contributing to search for novel diagnostic and therapeutic targets.

LncRNAs barely encode proteins, with over 200 nucleotides in length 9. Growing evidences have revealed the functions of lncRNAs in many biological processes through multiple levels 10. In particular, differentially expressed lncRNAs participate in tumor development, and can be utilized as diagnostic marker for predicting the risk of tumorigenesis 11,12. For example, lncAA174084 expression in gastric juice can predict the occurrence of gastric cancer 13. Serum level of lncRNA PCAT1 serves as a diagnostic marker for multiple myeloma 14. Some certain lncRNAs have been identified to be involved in the occurrence, progression and metastasis of bladder cancer. Imprinted gene lncRNA H19, which is silenced in healthy adults, is highly expressed in bladder cancer and regulates tumor proliferation 15. Besides, abnormally high expressions of HOTAIR and UCA1 participate in the occurrence of bladder cancer 16,17.

In this study, we analyzed the TCGA database and reported the highly expressed lnc00518 in bladder cancer. This study analyzed the correlation between lnc00518 expression and prognosis of bladder cancer patients. In addition, its *in vivo* and* in vitro* biological functions in bladder cancer were specifically elucidated.

## Materials and Methods

### TCGA data analysis

RNA-seq data of bladder cancer and paracancerous tissues in the TCGA database (https://cancergenome.nih.gov/) were downloaded. Expression difference was analyzed by edger function, and prognosis was analyzed by survival function. Expression level and survival curves of lnc00518 in bladder cancer of TCGA database were determined.

### Subjects and tissue samples

72 pairs of bladder cancer tissues and paracancerous tissues were obtained from patients with bladder cancer at the Department of Urology, the First Affiliated Hospital of Nanjing Medical University from January 2009 to January 2014. Patients were selected by the following inclusion criteria: (1) All patients underwent radical or partial cystectomy; (2) All patients did not receive any treatment before the surgery; (3) All collected tumor samples were confirmed as bladder urothelial carcinoma by postoperative pathology; (4) All patients had complete follow-up information; (5) All patients signed informed consent. In addition, the major exclusion criterion was as follows: (1) Patients received preoperative adjuvant treatment such as radiotherapy and chemotherapy; (2) Patients lack sufficient follow-up data. Samples were immediately frozen and stored in liquid nitrogen. This study was approved by the Ethics Committee of the First Affiliated Hospital of Nanjing Medical University. Clinical information of enrolled subjects was detailed in Table 1.

### Cell culture

Human bladder cancer cell lines 5637, 253J, J82, T24and normal urothelial cell line SV-HUC-1 were purchased from the Cell Bank, Chinese Academy of Sciences, Shanghai. SV-HUC-1 and J82 cells were cultured with RPMI-1640 containing 1% penicillin-streptomycin and 10% fetal bovine serum (FBS). 5637, 253J and T24 cells were cultured in DMEM containing 1% penicillin-streptomycin and 10% FBS. All cells were maintained in a 5% CO_2_, 37 °C cell incubator.

### Cell transfection

Transfection of small interfering RNA (si-lnc00518) and overexpression plasmid (pcDNA-lnc00518): 5637 and T24 cells in logarithmic period with a good viability were transfected with si-lnc00518, pcDNA-lnc00518 or negative control using Lipofectamine 2000. Transfection vectors were synthesized by GenePharma (Shanghai, China). Cells were harvested 48 h after transfection for other experiments.

Transfection of lentivirus: 5637 and T24 cells were transfected with LV3-pGLV-h1-GFP-puro vector containing either the lnc00518 knockdown (sh-lnc00518) or a negative control sequence (Empty vector) respectively. Pools of stable transductants were generated by selection using puromycin (4 μg/ml) for 2 weeks. Transfection lentiviruses were provided by GenePharma (Shanghai, China).

### qRT-PCR

Total RNA was extracted with TRIzol reagent (Invitrogen, USA) and their relative cDNA was synthesized by Primescript RT Reagent (TaKaRa, Japan). QRT-PCR were performed by using StepOne Plus Real-Time PCR system (Applied Biosystems, USA) with SYBR® Premix Ex Taq™ Reagent (TaKaRa, Japan). The following primers were used for qRT-PCR:

Lnc00518: Forward: 5′-AAGTGGCACCAGCCTCACT-3′,

Reverse: 5′-CGGCCAAGT TCTTTACCATC-3′;

MiRNA-101: Forward: 5′-TGGGCTACAGTACTGTGATA-3′,

Reverse: 5′-TGCGTGTCGTGGAGTC-3′;

EZH2: Forward: 5′-AATCAGAGTACATGCGACTGAGA-3′,

Reverse: 5′-GCTGTATCCTTCGCTGTTTCC-3′;

β-actin: Forward: 5′-ACTGGAACGGTGAAGGTGAC-3′,

Reverse: 5′-AGAGAAGTGGGGTGGCTTTT-3′;

U6: Forward: 5′-CTCGCTTCGGCAGCAGCACATATA-3′,

Reverse: 5′-AAATATGGAACGCTTCACGA-3′.

Fold changes in RNA expression were calculated using 2^-ΔΔCt^ method and normalized based on β-actin with ABI Step One Software version 2.1.

### Western blot

Total protein from cells or tissues was extracted using RIPA on ice and shaken for 30 min. Lysis was centrifuged at 4°C, 14000×g for 15 min. Protein sample was quantified using BCA protein concentration determination kit (Pierce, Rockford, Il, USA) and loaded for electrophoresis using 10% SDS-PAGE. After transferring on a PVDF membrane at 300 mA for 100 minutes, it was blocked in 5% skim milk for 2 hours, washed with TBST for 6 times (10 min each) incubated with primary antibodies at 4 °C overnight and secondary antibodies for 2 hours. Bands were exposed by ECL and analyzed by Image Software. Primary antibodies used here were antibodies against EZH2 and actin, and secondary antibodies used were anti-mouse and anti-rabbit, all provided by Cell Signaling Technology.

### Cell proliferation assay

After transfection for 48 h, cells were cultured in 96-well plates at a cell density of 1×10^6^/mL with 100 μL per well. Each sample set 5 replicate wells. After incubation for 24 h, 48 h, 72 h and 96 h, 10 μL of CCK-8 was added each well. Absorbance was recorded at 450 nm with a microplate reader for plotting the growth curve.

### Colony formation assay

Transfected cells for 48 hours were seeded in 6-well plates with 600 cells per well and cultured in complete medium for 2 weeks. Medium was changed every 3 days. After colony formation, they were subjected to fixation with 5% paraformaldehyde for 30 min and dye with 0.1% crystal violet for another 30 min. Colonies were washed with PBS and captured for counting.

### Cell cycle assay

Transfected cells for 48 hours were seeded in 6-well plates with 1×10^5^ cells per well. After incubation overnight, cells were subjected to fixation with ethanol at 4 °C overnight and dye with PI for 25 min. Finally, cell cycle progression was determined using flow cytometry.

### Transwell cell migration and invasion assay

Transfected cells for 48 hours were suspended in serum-free medium with 2.0×10^5^/ml. Transwell chamber with or without pre-coated Matrigel was inserted in the 24-well plates, containing 200 μL of suspension in the apical chamber and 500 μL of medium with 10% FBS in the basolateral chamber. At 48 h, chambers were taken out and penetrating cells were subjected to fixation with 5% paraformaldehyde for 30 min and dye with 0.1% crystal violet for 15 min. Penetrating cells were captured for counting with 5 randomly selected fields in each sample.

### Subcutaneous tumorigenesis in nude mice

Ten 5-week-old female BALB/C nude mice were randomly assigned into 2 groups for injection of differentially treated cell suspension, with 5 in each group. 5637 cells with stable knockdown of lnc00518 (sh-lnc00518) or controls (empty vector) were prepared for suspension and subcutaneously injected in the right upper limbs of nude mice. Tumor size was measured every three days. Nude mice were sacrificed 3 weeks later and tumors were harvested for immunohistochemistry. Animal procedures were approved by the Animal Research Ethics Committee of Nanjing Medical University.

### Dual-luciferase reporter gene assay

The wild-type and mutant-type lnc00518 sequences containing the binding site of miR-101 were inserted into the pmirGLO dual-luciferase vector and then construct the reporter vector lnc00518-wild type (lnc00518-WT) and lnc00518-mutant type (lnc00518-MUT). lnc00518-WT or lnc00518-MUT, and miR-101 mimics were co-transfected into the HEK293T cells by Lipofectamine 3000. Next, cells were harvested and luciferase activity was measured with Dual-luciferase Reporter Assay System at 48h, according to manufacturer′s procedure. Similarly, the EZH2-WT and EZH2-MUT reporter vectors were constructed follow the same procedure and the relative luciferase activities were detected as described above.

### RIP assay

RIP assay was performed using the Magna RIP RNA-Binding Protein Immunoprecipitation Kit (Millipore, USA). Cells with 80-90% of confluence were lysed using RIP lysis buffer and incubated with RIP buffer containing magnetic beads bound to human anti-Ago2 antibody or negative control IgG. Samples were incubated with proteinase K to digest the protein, followed by precipitation of the RNA. The purified RNA was finally subjected for qRT-PCR.

### Statistical analysis

SPSS 22.0 was used for all statistical analysis and GraphPad Prism 7.0 was used for figure editing. Data were represented as mean ± SD. The *t*-test was used for analyzing measurement data. Categorical data were analyzed using chi-square test. *P*<0.05 indicated the significant difference.

## Results

### Lnc00518 is highly expressed in bladder cancer

**Figure 1:**
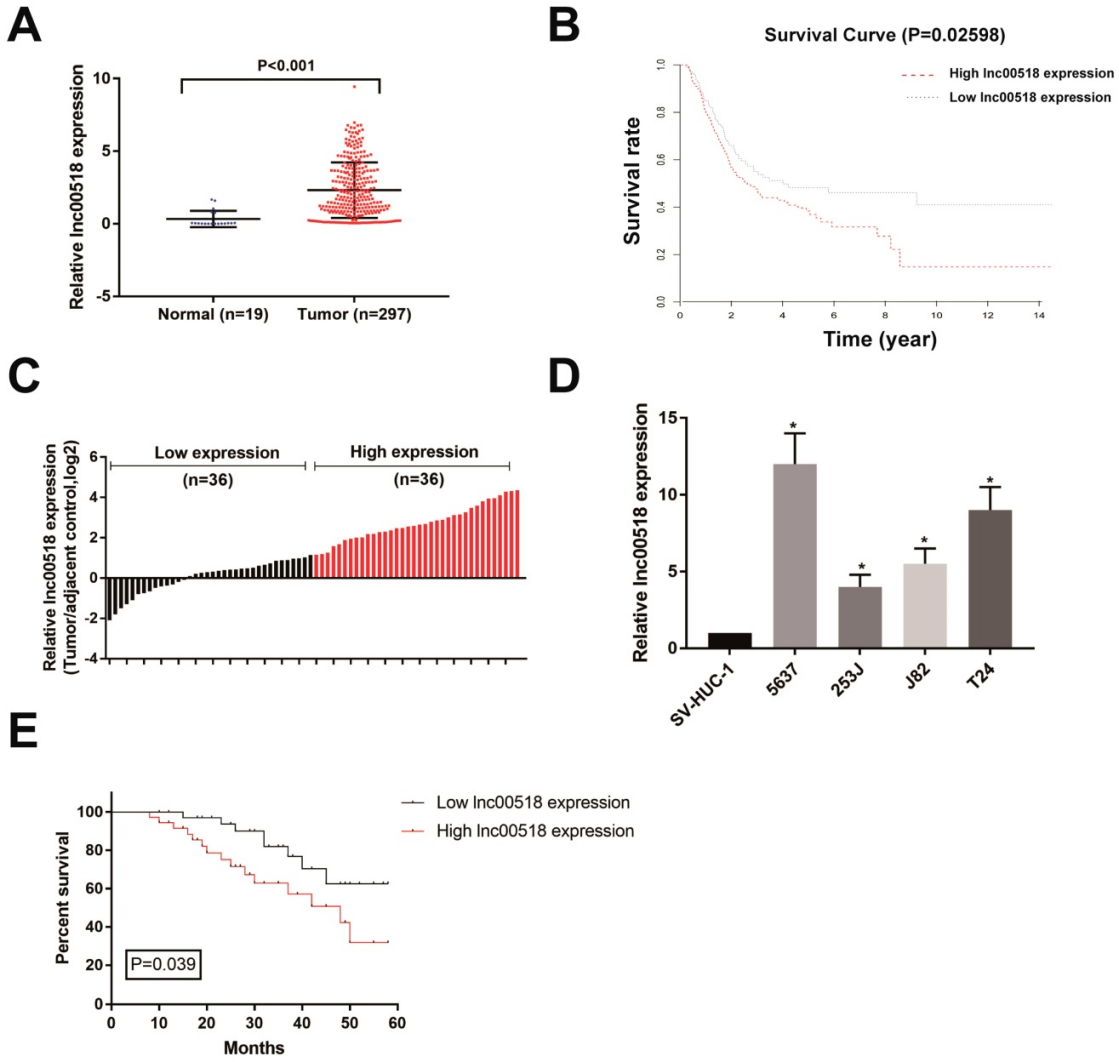
** Lnc00518 is highly expressed in bladder cancer. A.** TCGA database showed that lnc00518 expression was higher in bladder cancer tissues than paracancerous tissues. **B.** Survival analysis of TCGA database showed a negative correlation between lnc00518 expression with overall survival of bladder cancer. **C.** Lnc00518 was highly expressed in bladder cancer tissues than paracancerous tissues collected from our hospital. **D.** Lnc00518 expression was higher in bladder cancer cells than normal human urothelial cells. E. Survival analysis showed a negative correlation between lnc00518 expression with overall survival of bladder cancer based on the enrolled patients in our hospital.

Through analyzing TCGA database, lnc00518 expression was higher in bladder cancer tissues than paracancerous tissues (Figure 1A). Survival analysis showed a negative correlation between lnc00518 expression with overall survival of bladder cancer (Figure 1B). We then selected 72 pairs of bladder cancer tissues and paracancerous tissues in our hospital for detecting lnc00518 expression. Consistently, lnc00518 was highly expressed in bladder cancer tissues (Figure 1C). Subsequently, lnc00518 expression in bladder cancer cells and normal human urothelial cells were determined. Lnc00518 expression remained higher in bladder cancer cells, especially in 5637 and T24 cells (Figure 1D).

### Lnc00518 expression is correlated with histological grade, clinical stage and overall survival of bladder cancer patients

Based on lnc00518 expression, enrolled bladder cancer patients were assigned into high-expression and low-expression group. Correlation analysis was conducted using chi-square test for evaluating the relationship between lnc00518 expression with their clinical data. Lnc00518 expression was positively correlated with TNM staging and histological grade of bladder cancer, but not correlated with age, gender, lymph node metastasis and tumor number (Table 1). Follow-up data were collected for analyzing the prognosis of bladder cancer patients. Kaplan-Meier survival curve indicated that high expression of lnc00518 was associated with poor prognosis of bladder cancer (Figure 1E). The higher the expression level of lnc00518, the worse the prognosis of bladder cancer. We may conclude that lnc00518 was a novel diagnostic marker for bladder cancer.

### Lnc00518 regulates cellular behaviors of bladder cancer cells

**Figure 2:**
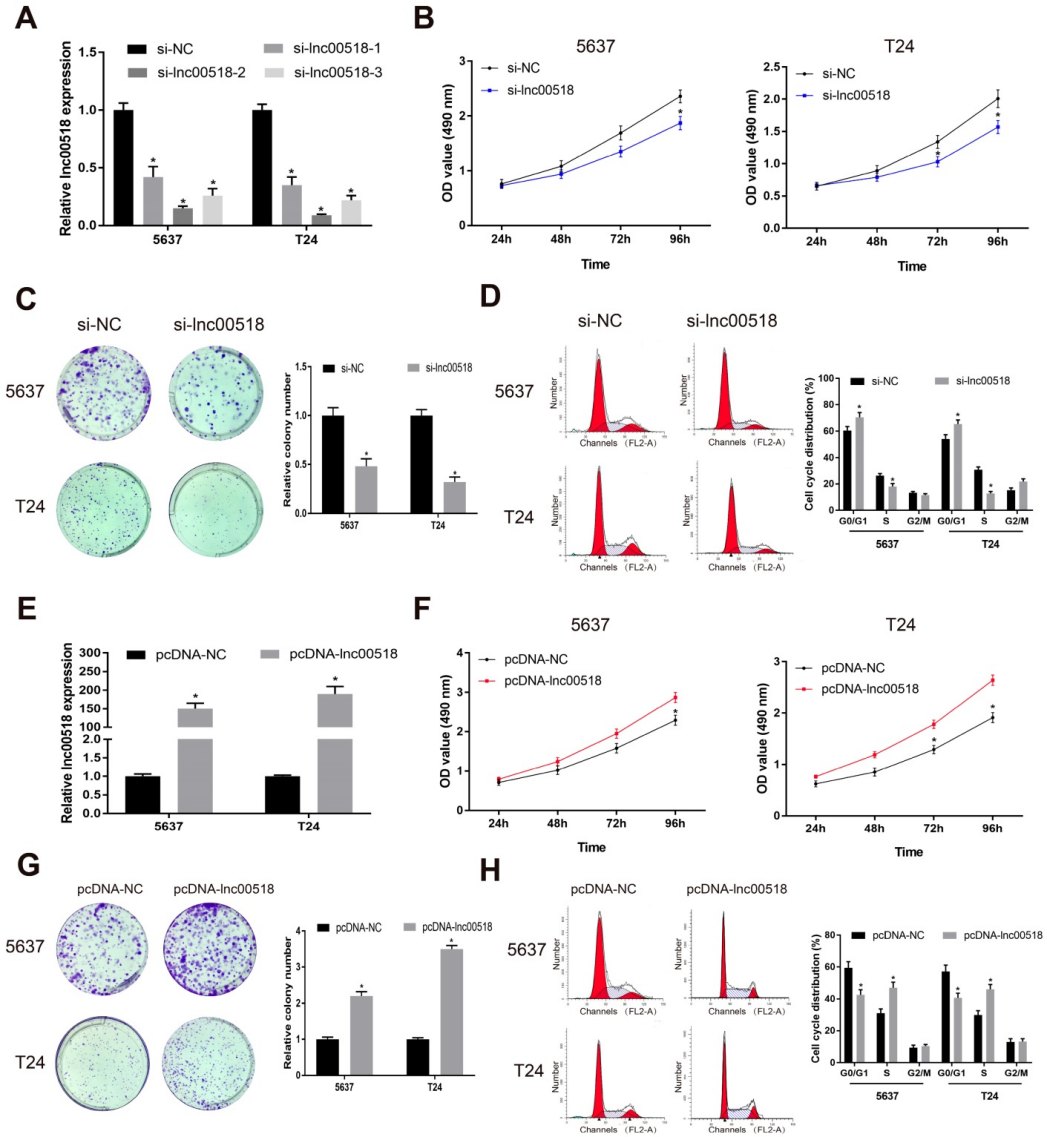
** Lnc00518 promotes the proliferation of bladder cancer cells. A.** Transfection efficacy of si-lnc00518-1, si-lnc00518-2 and si-lnc00518-3 in 5637 and T24 cells. **B-D.** 5637 and T24 cells were transfected with si-lnc00518 or si-NC, which were subjected to CCK-8 assay for determining cell viability **(B)**, colony formation assay for determining clonality **(C)** and flow cytometry for determining cell cycle progression **(D)**. **E.** Transfection efficacy of pcDNA-lnc00518 in 5637 and T24 cells. **F-H.** 5637 and T24 cells were transfected with pcDNA-lnc00518 or pcDNA-NC, which were subjected to CCK-8 assay for determining cell viability **(F)**, colony formation assay for determining clonality **(G)** and flow cytometry for determining cell cycle progression** (H)**. (* P < 0.05)

**Figure 3:**
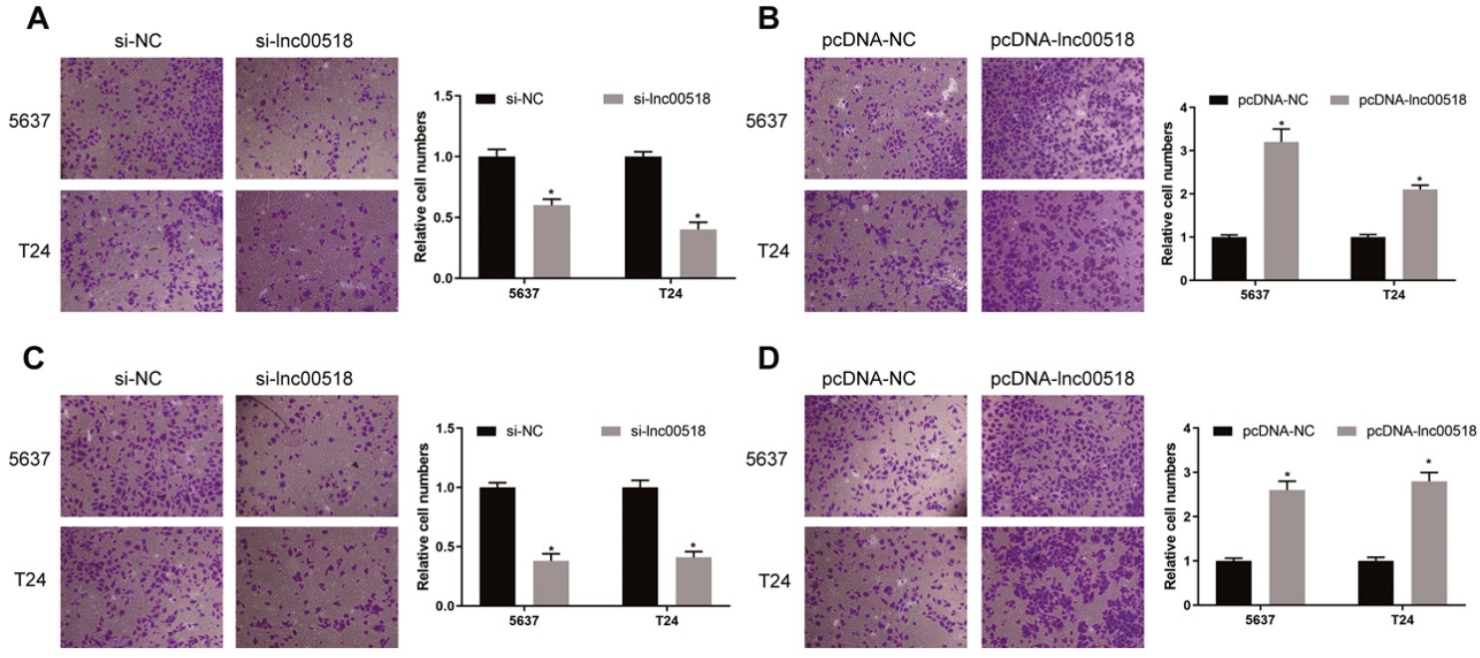
** Lnc00518 promotes the invasion and migration of bladder cancer cells. A.** Migration of 5637 and T24 cells transfected with si-lnc00518 or si-NC. **B.** Migration of 5637 and T24 cells transfected with pcDNA-lnc00518 or pcDNA-NC. **C.** Invasion of 5637 and T24 cells transfected with si-lnc00518 or si-NC. **D.** Invasion of 5637 and T24 cells transfected with pcDNA-lnc00518 or pcDNA-NC. (* P < 0.05)

To further explore the potential function of lnc00518 in bladder cancer, we successfully constructed si-lnc00518 and pcDNA-lnc00518. Transfection efficacy was verified in 5637 and T24 cells (Figure 2A, E). By transfection of si-lnc00518, proliferative rate and clonality were markedly reduced (Figure 2B, C). Conversely, 5637 and T24 cells overexpressing lnc00518 showed increased proliferative rate and clonality (Figure 2F, G). Lnc00518 knockdown elevated cell ratio in G1 phase, while overexpression of lnc00518 obtained the opposite results (Figure 2D, H). We believed that lnc00518 was capable of accelerating cell cycle progression and proliferative potential of bladder cancer cells. In addition, Transwell assay proved the promotive role of lnc00518 in migratory and invasive potentials of 5637 and T24 cells (Figure 3).

### Lnc00518 promotes tumorigenesis *in vivo*

**Figure 4:**
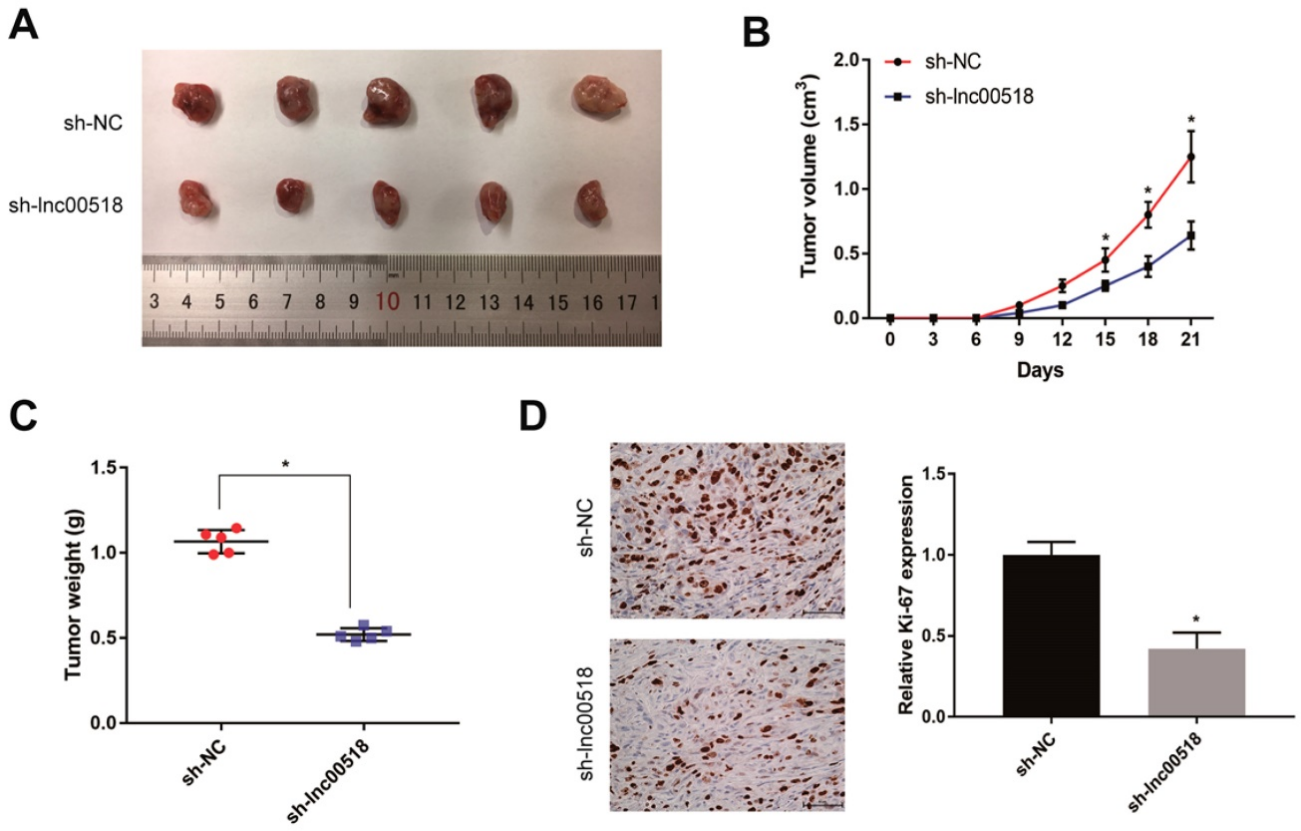
** Lnc00518 promotes* in vivo* tumorigenesis of bladder cancer.** 5637 cells transfected with sh-lnc00518 or empty vector were prepared for suspension and subcutaneously injected in the right upper limbs of nude mice. **A.** Bladder cancer tissues harvested from nude mice. **B.** Tumor volume (cm^3^). **C.** Tumor weight (g). **D.** Immunohistochemical staining of Ki-67. (* P < 0.05)

To further validate the effect of lnc00518 on bladder cancer, 5637 cells transfected with sh-lnc00518 or empty vector were prepared for suspension and subcutaneously injected in the right upper limbs of nude mice. Mice with lnc00518 knockdown presented slower tumor growth, lower tumor volume and tumor weight than controls (Figure 4A-C). Immunohistochemistry results suggested that lnc00518 knockdown inhibited positive expression of Ki-67, indicating the inhibited *in vivo* proliferation of bladder cancer (Figure 4D).

### Lnc00518 directly interacts with miRNA-101

**Figure 5:**
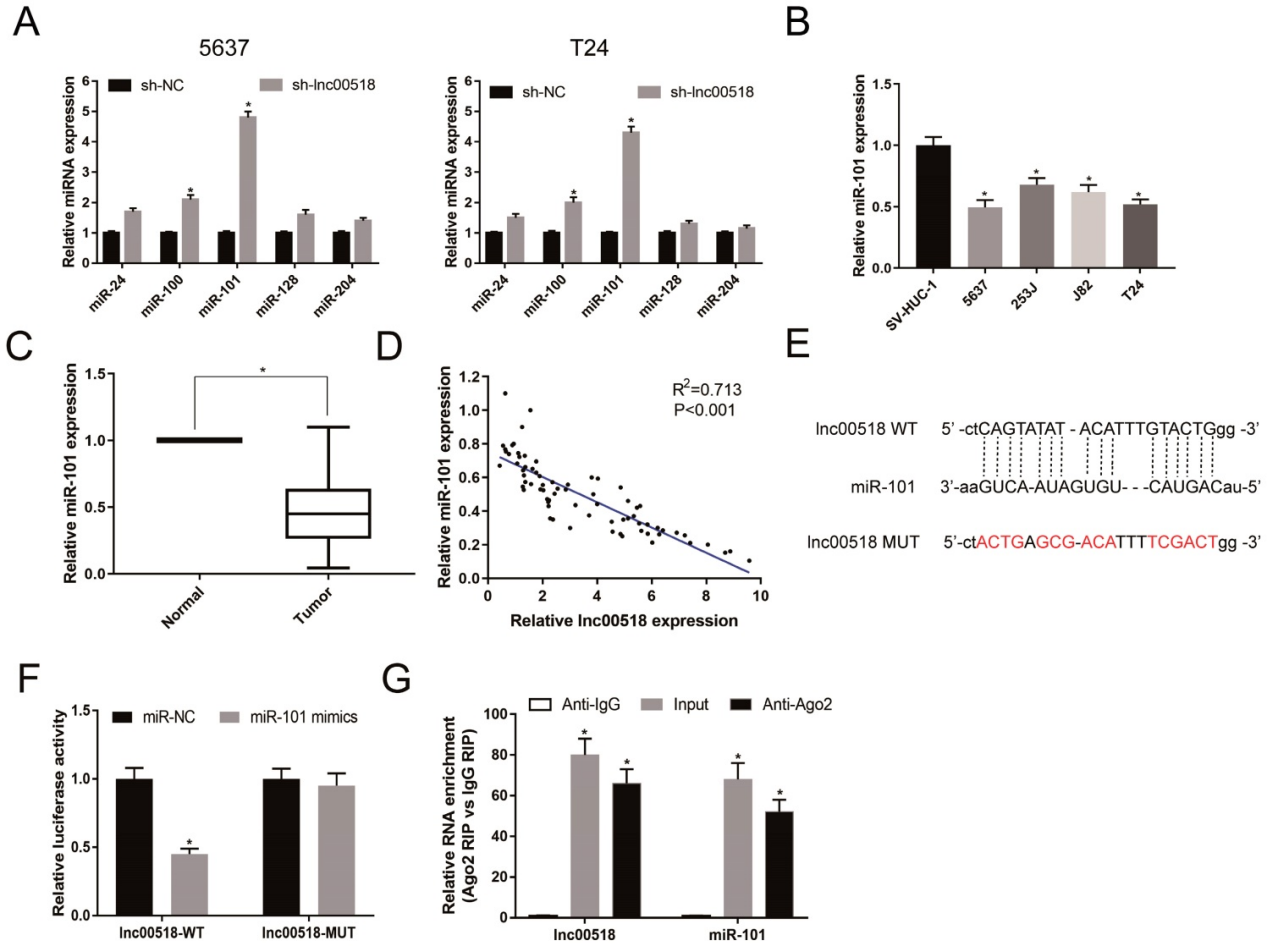
** Lnc00518 directly interacts with miR-101. A.** MiR-24, miR-100, miR-101, miR-128 and miR- 204 exerted potential binding sites with lnc00518, which were all upregulated by lnc00518 knockdown, and miR-101 was the most upregulated one. **B, C**. MiR-101 was lowly expressed in bladder cancer cells** (B)** and tissues **(C)**. **D.** MiR-101 expression was negatively correlated with lnc00518 expression. **E.** Binding sites between lnc00518 and miR-101. **F.** Co-transfection of miR-101 mimics and lnc00518-WT markedly decreased the luciferase activity, whereas cells co-transfected with miR-101 mimics and lnc00518-MUT did not present obvious change in luciferase activity. **G.** Increased abundances of lnc00518 and miR-101 in the Ago2 antigen-antibody complex. (* P < 0.05)

Through searching for possible downstream genes of lnc00518 in RegRNA, we found that miR-24, miR-100, miRNA-101, miR-128 and miR- 204 exerted potential binding sites with lnc00518. These predicted genes were all upregulated by lnc00518 knockdown, and miRNA-101 was the most upregulated one (Figure 5A). Next, miRNA-101 expression was found to be lowly expressed in bladder cancer cells and tissues (Figure 5B, C). The data from GSE112264 also showed miR-101 was down-regulated in bladder cancer tissues (Figure S1A). A negative correlation was identified between miRNA-101 expression and lnc00518 expression (Figure 5D). As a result, we speculated that lnc00518 may target miRNA-101 to exert its biological function.

Based on the binding sites between lnc00518 and miRNA-101, lnc00518-WT and lnc00518-MUT were constructed (Figure 5E). Co-transfection of miRNA-101 mimics and lnc00518-WT markedly decreased the luciferase activity, whereas cells co-transfected with miRNA-101 mimics and lnc00518-MUT did not present obvious change in luciferase activity (Figure 5F). Moreover, abundances of lnc00518 and miRNA-101 in the Ago2 antigen-antibody complex remarkably increased, indicating that lnc00518 and miRNA-101 could bind to Ago2 (Figure 5G). The above data all demonstrated that lnc00518 directly interacted with miRNA-101 as a ceRNA.

### EZH2 was the downstream target for miRNA-101

**Figure 6:**
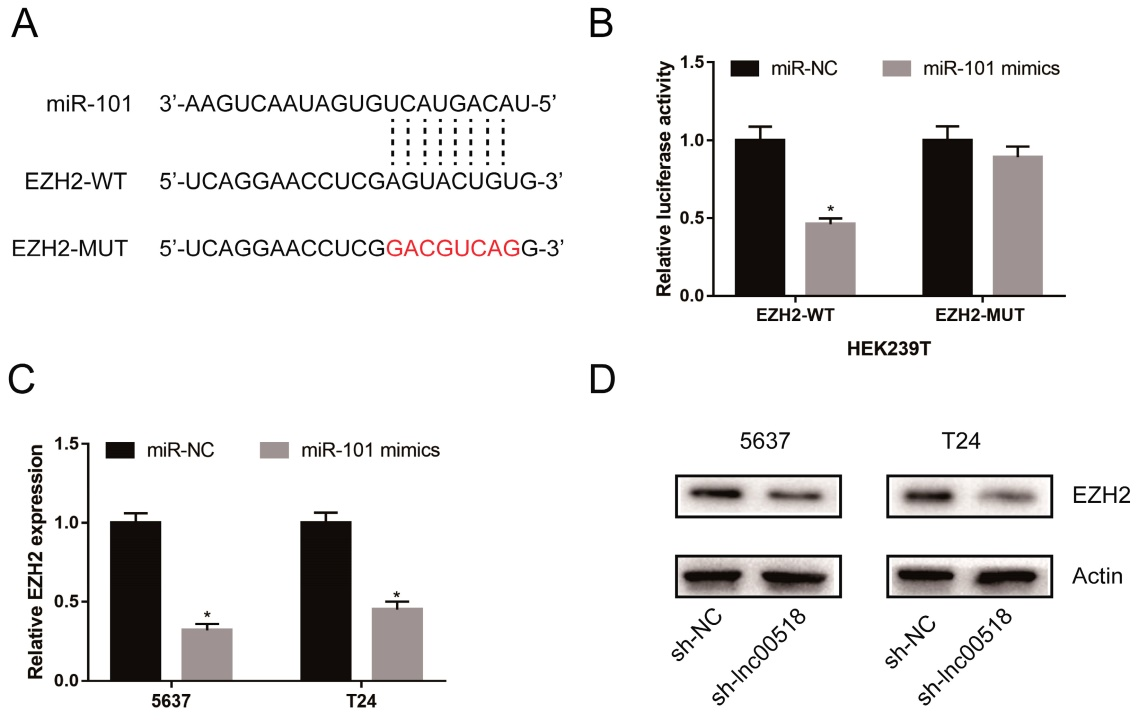
** EZH2 was the downstream target for miR-101. A.** Binding sites between miR-101 and EZH2. **B.** Dual-luciferase reporter gene assay showed that luciferase activity markedly decreased in cells co-transfected with miR-101 mimics and EZH2-WT, while it was not altered in those co-transfected with miR-101 mimics and EZH2-MUT. **C.** Overexpression of miR-101 significantly reduced the expression of EZH2. **D.** Down-regulating lnc00518 decreased the expression of EZH2. (* P < 0.05)

Through analyzing online bioinformatics database (TargetScan, miRDB, and miRTarBase), we found that EZH2 was a potential downstream target for miRNA-101. In TCGA database, we found EZH2 was up-regulated in bladder cancer tissues (Figure S1B). Dual-luciferase reporter gene assay showed that luciferase activity markedly decreased in cells co-transfected with miRNA-101 mimics and EZH2-WT, while those co-transfected with miRNA-101 mimics and EZH2-MUT showed unchangeable luciferase activity (Figure 6A, B). After overexpression of miR-101 in 5637 and T24 cells, the expression of EZH2 was significantly decreased, suggesting that miR-101 can negatively regulate EZH2 (Figure 6C). Lnc00518 expression was positively correlated with EZH2 expression in TCGA database (Figure S1C). In addition, Western Blot results showed that the expression of EZH2 decreased after down-regulation of lnc00518 expression (Figure 6D). We believed that there was a presence of lnc00518/miRNA-101/EZH2 axis involving in the pathogenesis of bladder cancer.

### Lnc00518 regulated behaviors of bladder cancer cells *via* miRNA-101/EZH2

**Figure 7:**
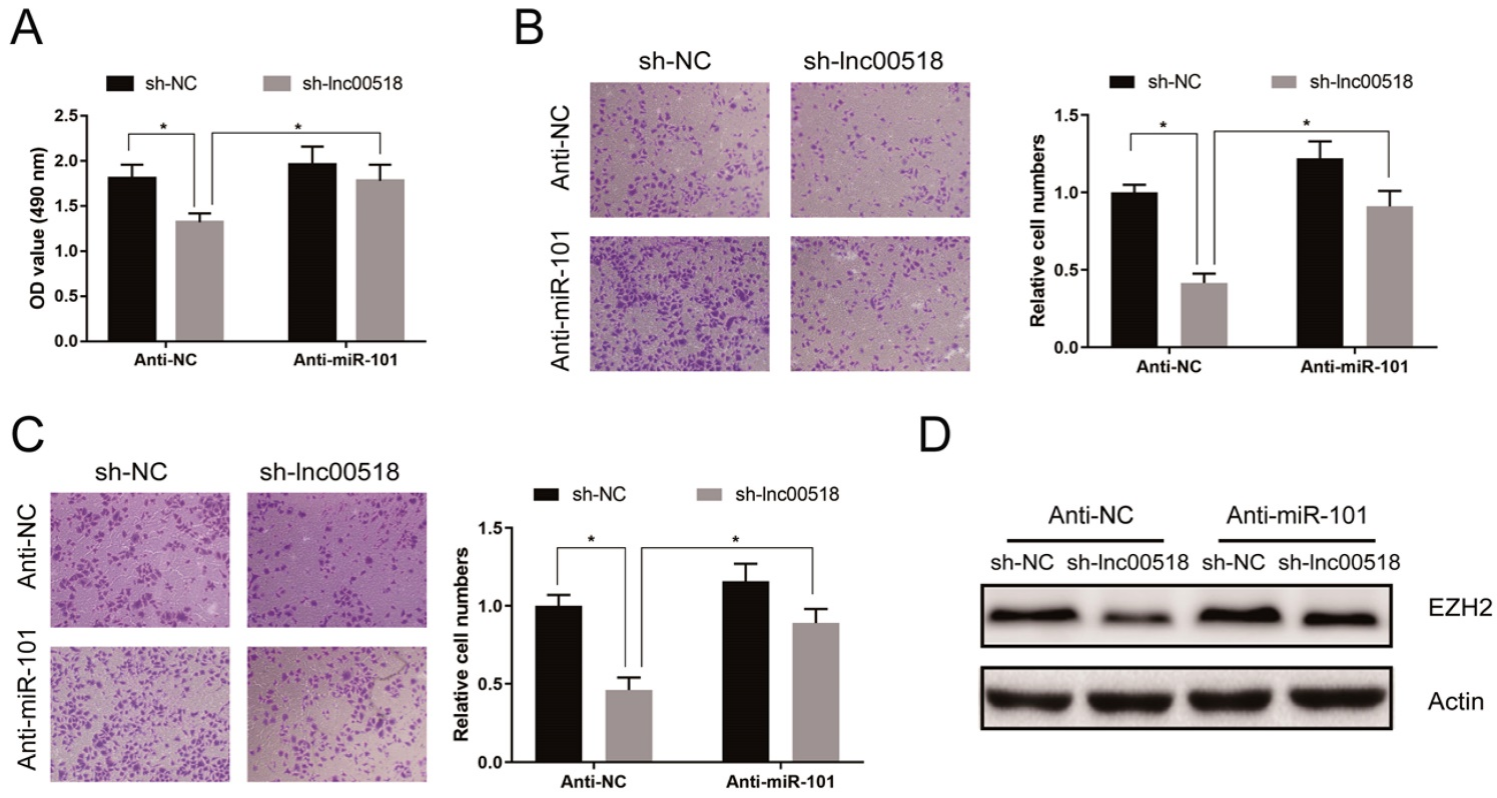
** Lnc00518 regulated behaviors of bladder cancer cells *via* miR-101/EZH2. A.** CCK-8 assay showed that miR-101 knockdown could reverse the decreased proliferation induced by lnc00518 knockdown. **B, C.** Inhibited migratory **(B)** and invasive **(C)** potentials of 5637 cells due to lnc00518 knockdown were partially reversed by miR-101 knockdown. **D.** Downregulation of EZH2 expression caused by lnc00518 knockdown was reversed by inhibition of miR-101 expression. (* P < 0.05)

Rescue experiments were conducted to elucidate the role of lnc00518/miRNA-101/EZH2 axis in bladder cancer. CCK-8 assay showed that miRNA-101 knockdown could reverse the decreased proliferation induced by lnc00518 knockdown (Figure 7A). Similarly, inhibited migratory and invasive potentials of 5637 cells due to lnc00518 knockdown were partially reversed by miRNA-101 knockdown (Figure 7B, C). Besides, downregulation of EZH2 expression caused by lnc00518 knockdown was reversed by miRNA-101 silence (Figure 7D).

## Discussion

In recent years, a large number of studies have shown the significance of lncRNA in tumor development. With the development of sequencing technology, many new tumor-associated lncRNAs have emerged 11. Based on their functions in tumor development, lncRNAs could be divided into tumor-promoting factors, tumor-suppressor factors and tumor-promoting/tumor-suppressor factors 18. However, their specific functions in tumors still need to be comprehensively explored.

This study screened out lnc00518 from TCGA, which was highly expressed in bladder cancer. Survival analysis showed that high expression of lnc00518 was negatively correlated with the overall survival of bladder cancer patients. Identically, lnc00518 was highly expressed in bladder cancer cells. By analyzing the clinical data of enrolled patients, it is suggested that high expression of lnc00518 was correlated with TNM staging and histological grade of bladder cancer. These data indicated a tumor-promoting role of lnc00518 in bladder cancer.

To further explore the potential function of lnc00518 in bladder cancer, we constructed cell model with stable overexpression or knockdown of lnc00518. Lnc00518 overexpression enhanced proliferative, invasive, migratory potentials and clonality, but shortened G0/G1 phase of bladder cancer cells. Lnc00518 knockdown obtained the opposite trends. *In vivo* experiments revealed that lnck00518 knockdown inhibited subcutaneous tumorigenesis in nude mice.

Recent research have proposed that lncRNA can function as a natural miRNA sponge to regulate miRNA expression 19,20. For example, lncRNA PVT1 serves as a ceRNA in gastric cancer to sponge miR-186, thus promoting proliferative and invasive rates of gastric cancer cells 21. However, relationship between lncRNA and miRNA in bladder carcinogenesis is rarely reported. By bioinformatics analysis, miRNA-101 was screened as a target for lnc00518, and its expression was negatively regulated by lnc00518. Furthermore, we found that miRNA-101 was downregulated in bladder cancer cells, suggesting that lnc00518 directly degraded miRNA-101 expression. Dual-luciferase reporter gene assay verified the binding relationship between lnc00518 to miRNA-101. More importantly, RIP assay demonstrated an endogenous interaction between lnc00518 and miRNA-101, which was proved by their great abundances in Ago2 antibody.

MicroRNAs exert vital roles in promoting or inhibiting tumor development 22,23. It is reported that miRNA-101 is downregulated in many malignancies, including colorectal cancer, non-small cell lung cancer, ovarian cancer and bladder cancer 24-26. Our study proved that miRNA-101 expression was lowly expressed in bladder cancer tissues and cells, which was consistent with previous studies. EZH2 was searched from online bioinformatics database as a potential downstream target for miRNA-101. As an epigenetic regulator, EZH2 is highly expressed in a variety of malignant tumors and associated with poor prognosis of tumors including colorectal cancer,bladder cancer, non-small cell lung cancer 27-29.

We confirmed the binding relationship between miRNA-101 and EZH2 by dual-luciferase reporter gene assay. Overexpression of miRNA-101 in 5637 and T24 cells downregulated EZH2 expression, suggesting that miRNA-101 negatively regulated EZH2. In addition, lnc00518 knockdown was capable of downregulating EZH2 expression, indicating a presence of lnc00518/miRNA-101/EZH2 axis in the regulation of bladder cancer. Rescue experiments were conducted to reveal the potential role of lnc00518/miRNA-101/EZH2 axis. It is found that miRNA-101 knockdown reversed the inhibitory roles of lnc00518 knockdown in proliferative, migratory and invasive potentials of bladder cancer cells. EZH2 downregulation induced by lnc00518 knockdown was also reversed by miRNA-101 knockdown.

In conclusion, lnc00518 is highly expressed in bladder cancer and can be served as a predictor of poor prognosis. Lnc00518 promotes the proliferative, invasive and migratory potentials of bladder cancer by competitively binding to miR-101 to upregulate EZH2 expression.

## Supplementary Material

Supplementary figure.

## Figures and Tables

**Table 1:** Correlation between lnc00518 expression with pathological characteristics of bladder cancer patients (n=72).

Parameters	Number of cases	lnc00518 expression		P -value
		Low	High	
Age (years)				0.448
<60	23	13	10	
≥60	49	23	26	
Gender				0.276
Male	54	25	29	
Female	18	11	7	
TNM stage				**0.016**
Ta,T1	28	19	9	
T2-T4	44	17	27	
Histological grade				**0.009**
Low	35	23	12	
High	37	13	24	
Lymph nodes metastasis				0.075
No	63	34	29	
Yes	9	2	7	
Multiplicity				0.093
Single	43	25	18	
Multiple	29	11	18	
