# Supplementary material for: LncRNA00518 promotes cell proliferation through regulating miR-101 in bladder cancer
Source: J Cancer. 2020 Jan 14;11(6):1468–77. doi: 10.7150/jca.35710 (PMC6995372; doi:10.7150/jca.35710)
Supplement: Supplementary file 1 — Supplementary figure. [file jcav11p1468s1.pdf]

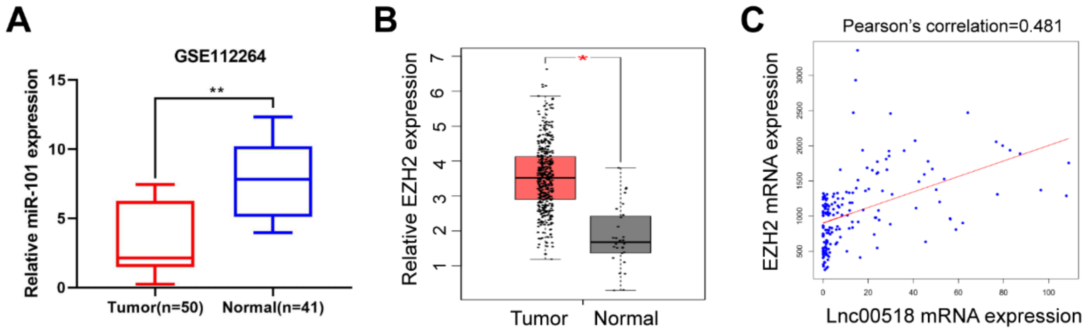

Figure S1. A. miRNA-101 was lowly expressed in bladder cancer tissues in GSE112264 database; **B.** EZH2 was up-regulated in bladder cancer tissues in TCGA database; **C.** Lnc00518 expression was positively correlated with EZH2 expression in TCGA database. （* $P < 0.05$,**$P < 0.01$）
